# Supplementary material for: Dynamic changes in large-scale functional connectivity prior to stimulation determine performance in a multisensory task
Source: Front Syst Neurosci. 2025 Feb 12;19:1524547. doi: 10.3389/fnsys.2025.1524547 (PMC11860953; doi:10.3389/fnsys.2025.1524547)
Supplement: Supplementary file 1 [file Data_Sheet_1.pdf]

Dynamic changes in large-scale functional connectivity prior to stimulation determine performance in a multisensory task

Edgar E. Galindo-Leon, Karl J. Hollensteiner, Florian Pieper,

Gerhard Engler, Guido Nolte, Andreas K. Engel

(Supplement)

Tables

Table 1. Statistics for frequency resolved power analysis for hit and miss trials. The table shows *p*-values for all *t*-tests within frequency bands, trial outcome, and across all time points of interest; *italics* indicate significant comparisons (p<0.001; FDR corrected).

Frequency	Theta	Alpha	Beta	Gamma	High-Gamma
	Baseline vs. stimulus onset window				
Hit vs. hit	0.22	0.26	0.44	*<0.001*	0.053
Miss vs. miss	0.23	0.54	0.99	*<0.001*	*<0.001*
	Stimulus onset vs. response onset window				
Hit vs. hit	0.03	0.18	0.08	*<0.001*	*<0.001*
Miss vs. miss	0.21	0.62	0.53	0.08	*<0.001*
	Response onset window vs. baseline				
Hit vs. hit	0.10	0.46	0.98	0.01	*<0.001*
Miss vs. miss	0.25	0.58	0.79	*<0.001*	*<0.001*

Table 2. Statistics for frequency resolved functional connectivity analysis for all time points of interest for hit and miss trials. The table shows *p*-values for all *t*-tests within frequency bands, trial outcome and across all time points of interest; *italics* indicate significant contrasts (FDR corrected).

Frequency	Theta	Alpha	Beta	Gamma	High-Gamma
	Baseline vs. stimulus onset window				
Hit vs. hit	0.12	*<0.001*	*<0.001*	*<0.001*	*<0.001*
Miss vs. miss	0.75	*<0.001*	*<0.001*	*<0.001*	*<0.001*
	Stimulus onset vs. response onset window				
Hit vs. hit	0.03	*<0.001*	0.36	*<0.001*	*<0.001*
Miss vs. miss	0.79	0.07	0.67	0.17	*<0.001*
	Response onset window vs. baseline				
Hit vs. hit	*<0.001*	0.02	0.003	*<0.001*	*<0.001*
Miss vs. miss	0.84	*<0.001*	*<0.001*	*<0.001*	*<0.001*

Figures

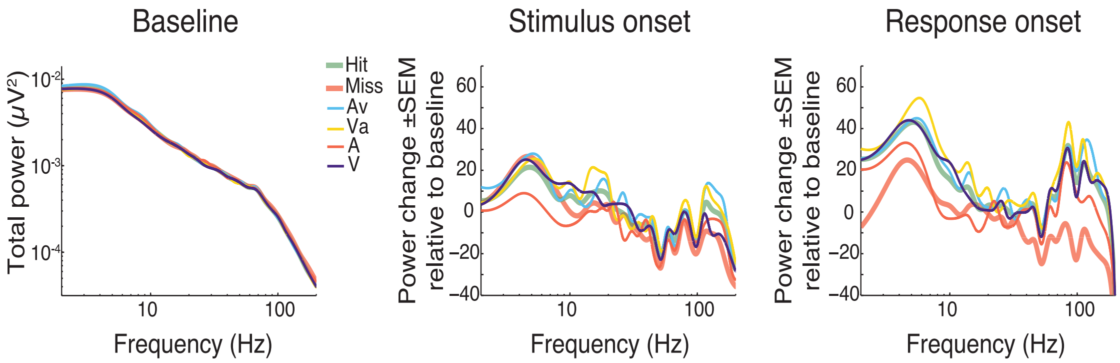

Figure S1. Left: total power in baseline intervals for each condition and response. Middle and right plots show the power change relative to baseline and response onset, respectively, for each condition.

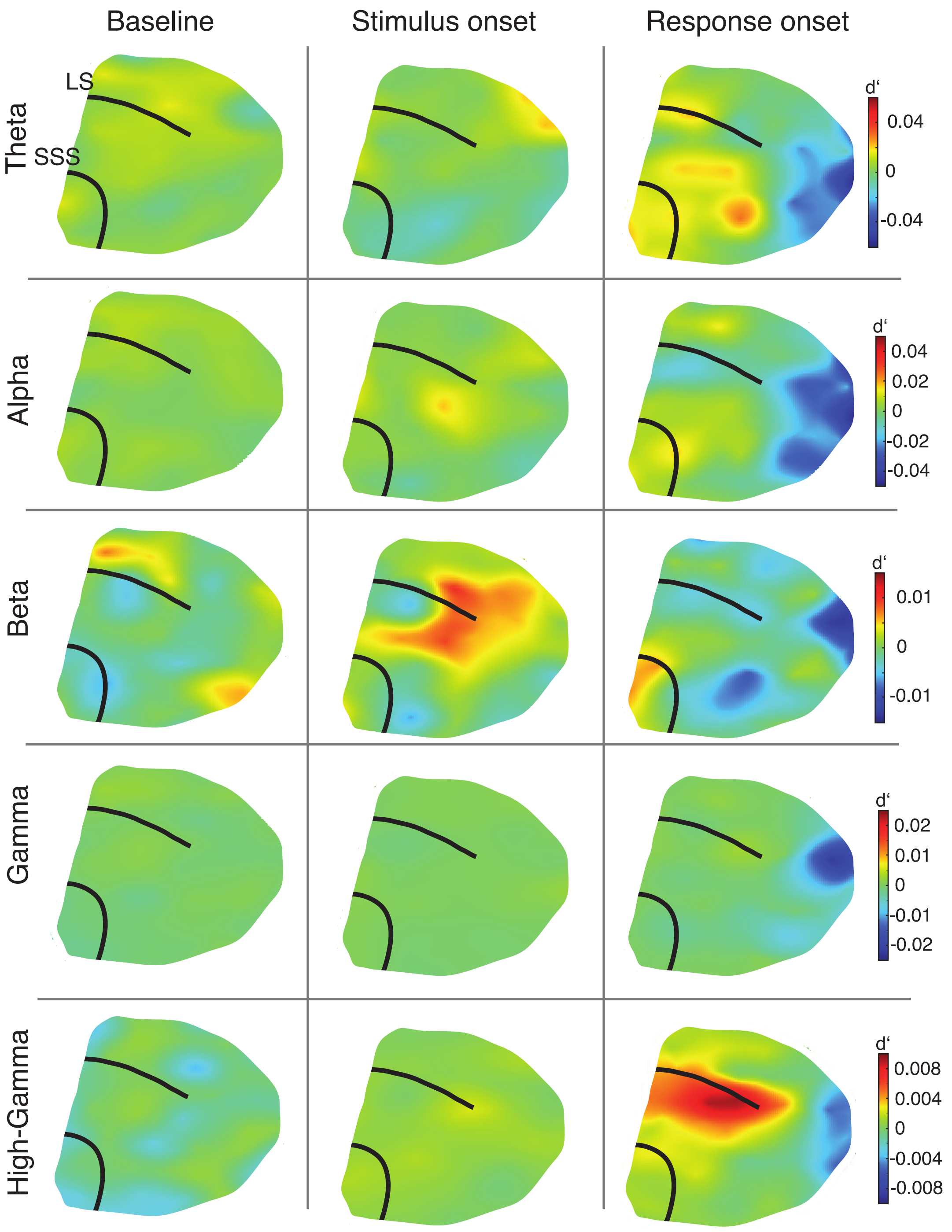

Figure S2. Topographic distribution of spectral differences between hit and miss trials for three different time windows (columns) and frequency bands (rows).

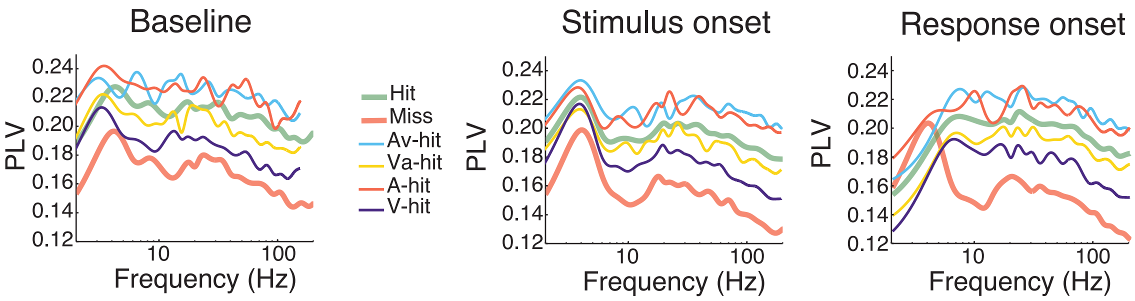

Figure S3. Effects of stimulus conditions and response on phase coupling (phase-locking value) in three different time windows. In contrast to power (cf. Fig. S1), differences in connectivity are observed in the baseline period.

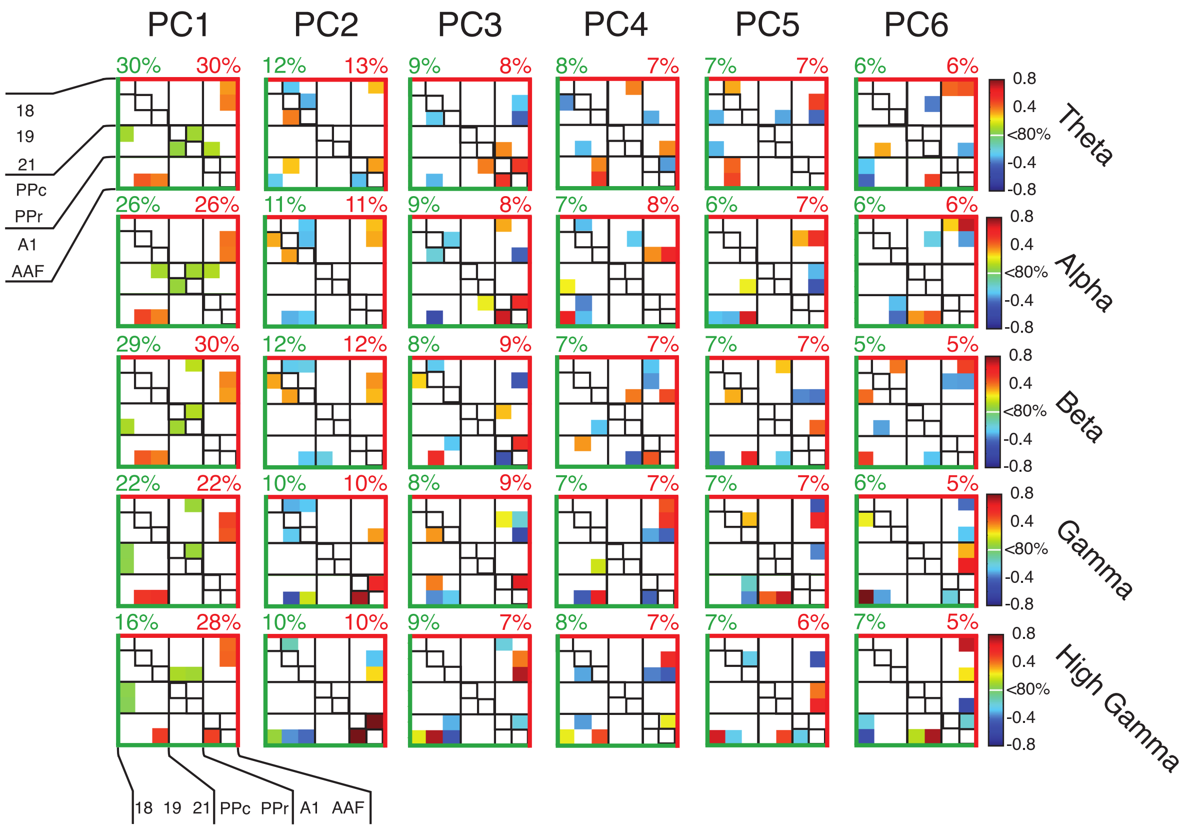

Figure S4. Eigenconnectivity patterns for hit trials (low triangle – green) and miss trials (upper triangle – red). Displayed are the connections that became stronger (hot colors) or weaker (cold colors) relative to the mean connectivity.

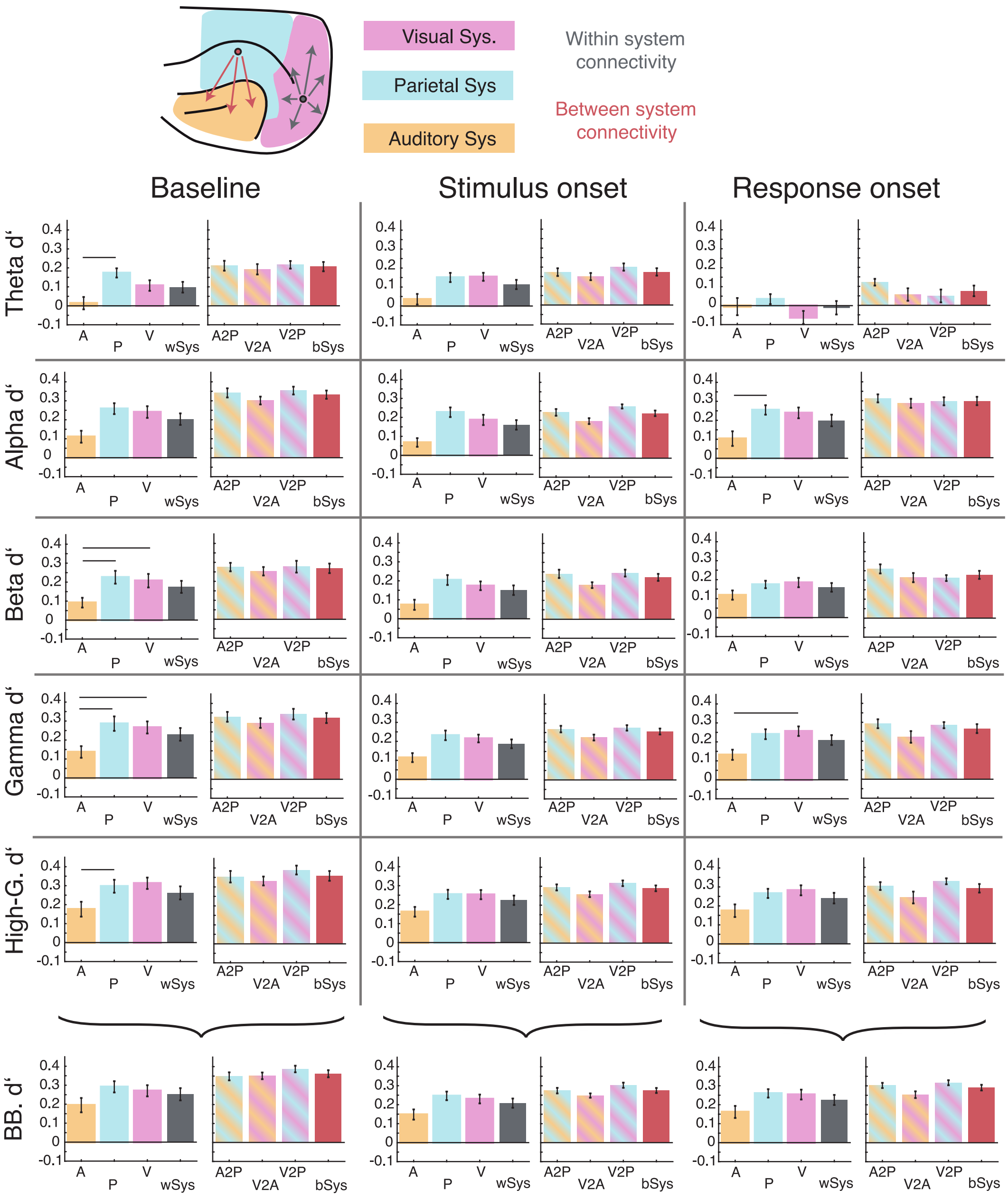

Figure S5. Contrast of within-system (full colors) and between-system (patterned colors) interactions. Bar patterns in the between-system conditions are composed of the colors of the individual systems shown on the left in each panel. Horizontal lines indicate significant differences ($p < 0.05$). See main text for details.

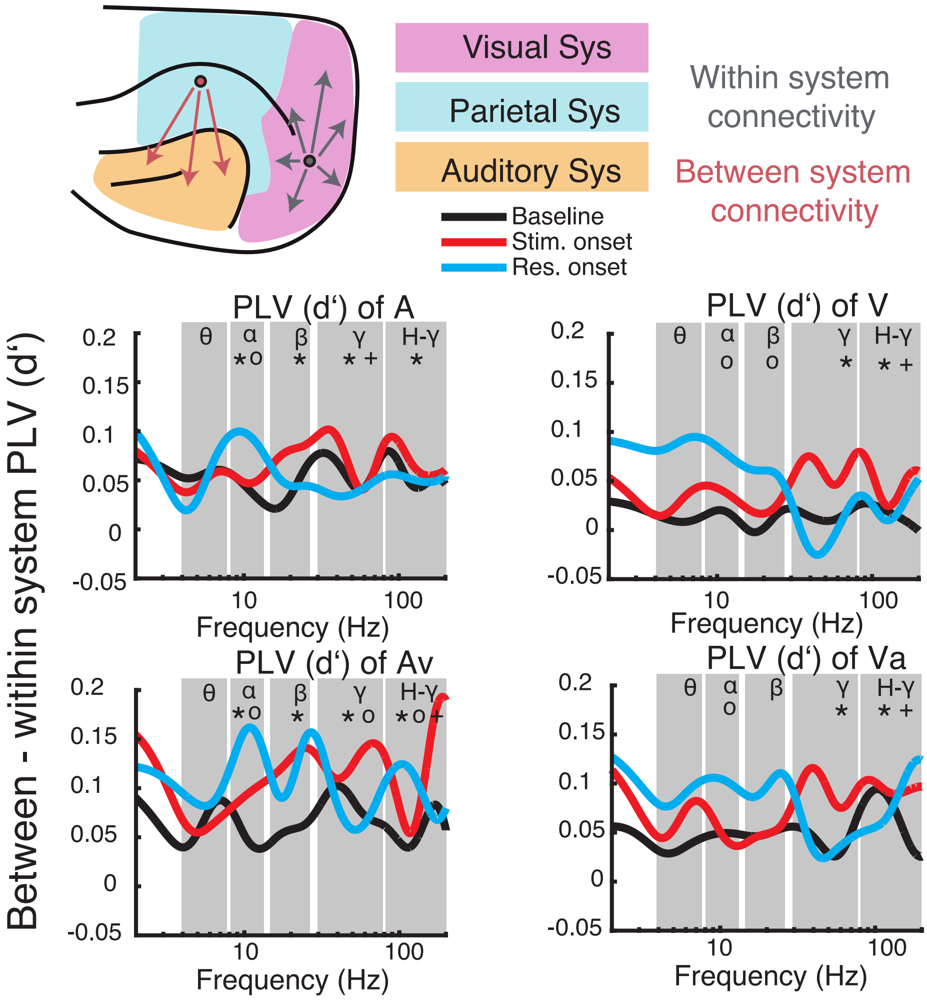

Figure S6. Differences in PLV (in d’) between long-range and short-range interactions (between and within systems, respectively).
